# Adult-Onset Woakes' Syndrome: Report of a Rare Case

**DOI:** 10.1155/2015/857675

**Published:** 2015-03-10

**Authors:** U. Schoenenberger, A. J. Tasman

**Affiliations:** Department of Otorhinolaryngology, Cantonal Hospital St. Gallen, Rorschacher Strasse 95, 9007 St. Gallen, Switzerland

## Abstract

*Introduction*. Woakes' syndrome, commonly defined as severe recurrent nasal polyps with consecutive destruction of the nasal pyramid, is rare with only a few reports in the literature documenting surgical treatment of the external nose. *Case Presentation*. We describe the case of an adult patient with Samter's triad who had been surgically treated from nasal polyposis since 2002. By 2014 a conspicuous deformity of the nasal pyramid had progressively occurred due to a recurrence. The patient underwent revision endoscopic sinus surgery and narrowing of the bony nasal vault by digital compression without osteotomies. *Discussion*. Having been described over 130 years ago, the etiology of Woakes' syndrome remains poorly understood. Treatment includes endoscopic sinus surgery and topical treatment. Surgical treatment of the external nose deformity by rhinoplasty is rarely addressed. *Conclusion*. This case illustrates that the widening of the bony nasal vault may be successfully corrected by digital compression, if the nasal bones are substantially thinned, in combination with surgical treatment of nasal polyps.

## 1. Introduction

Nasal polyps are a common entity which can lead to Woakes' syndrome, a very rare condition of nasal deformity due to extensive polyp growth in the paranasal sinuses and nasal cavity [1]. According to the literature, the syndrome is prevalent in children with the precise etiology being unknown. The treatment of the external nose in these cases has rarely been addressed so far.

## 2. Case Report

A 29-year-old female patient had first reported to the ENT Department of the Cantonal Hospital in St. Gallen, Switzerland, in 2001, with severe and progressive nasal blockage. Symptoms had begun 5 years earlier but had been left untreated. She was however known to have an aspirin and histamine intolerance. In addition, she had suffered from symptoms that had been interpreted as hay fever and asthma since her youth and reported a positive family history for nasal polyposis.

Physical findings documented at the time included a normal appearing external nose, complete bilateral obstruction of the nasal cavities by nasal polyps, and anosmia. The anosmia was determined by testing with Sniffin' sticks whereupon the patient was able to identify 3 out of 12 possible odors.

A CT scan showed hypoplastic frontal sinuses and a complete obstruction of all the other paranasal sinuses, with normal bony anatomy of the external nose. After medical treatment with topical and systemic steroids failed, the patient underwent an endoscopic sphenoethmoidectomy and maxillary sinus fenestration in 2002. Upon histologic examination of the polyp tissue respiratory mucosa with severe chronic eosinophilic inflammation was found. Postoperative treatment included topical and systemic corticosteroids. Aspirin desensitisation therapy was repeatedly discussed but declined by the patient. The sense of smell returned and the patient now identified correctly 11 out of 12 possible odors. The patient was free of recurrence during 5 months of follow-up in the hospital before continuing follow-up in the vicinity of her home. During this further follow-up, the sense of smell was not documented.

In March 2014, the 38-year-old patient was again referred to the ENT Department of Cantonal Hospital in St. Gallen, Switzerland, because of complete bilateral nasal obstruction with polyps visible in both nares and a progressive widening of the bony and cartilaginous nose. The patient reported a recurrence of nasal obstruction and a loss of sense of smell over the last 2-3 years and an increasingly noticeable nasal deformity in the last 12 months. Also pressure was felt over the maxillary but not frontal sinuses frequently. Postnasal drip symptoms were present in most days. Nasal treatment consisted of sporadic application of topical mometasone spray. Bronchial asthma symptoms had been stable with regular inhalation with formoterol and budesonide and regular systemic check-ups had shown no sign of bronchiectasis.

**Figure 1:**
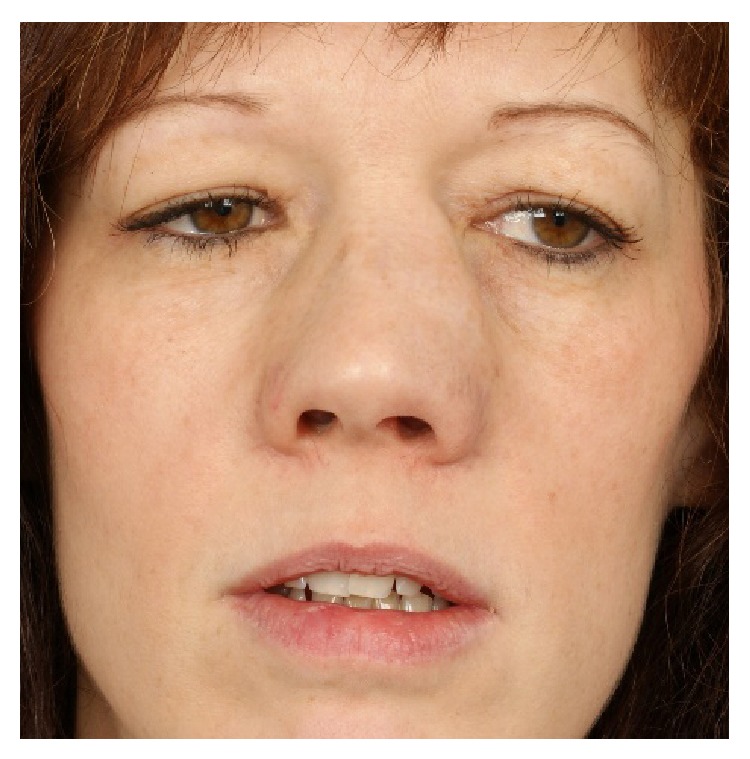
Nasal deformity at presentation (2014).

**Figure 2:**
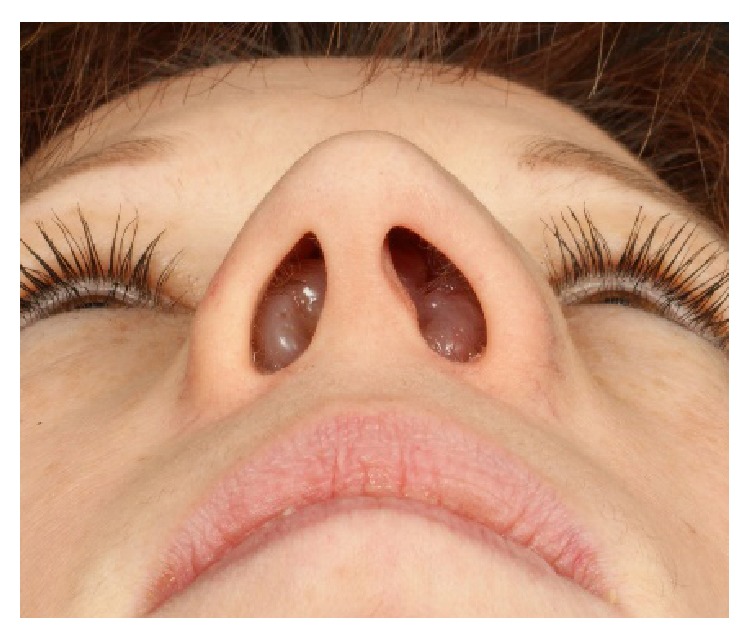
Massive nasal polyps at presentation (2014).

**Figure 3:**
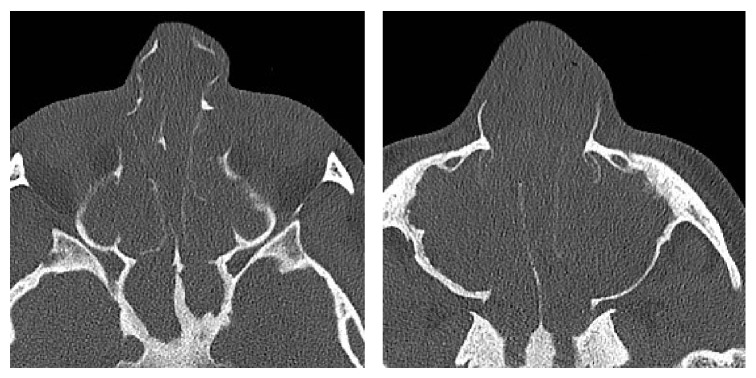
CT scan showing severe atrophy and deformity of the nasal pyramid.

**Figure 4:**
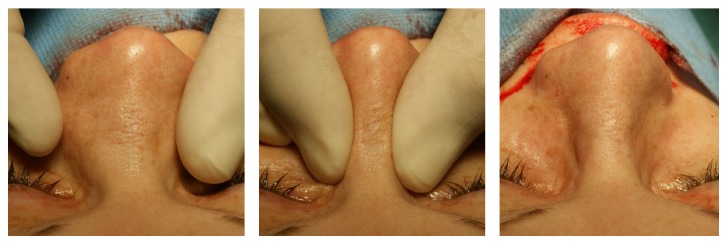
Intraoperative narrowing of the bony nasal vault by forced digital compression.

Clinical examination showed a distinct broadening and bilateral enlargement of the nasal pyramid (Figure 1). Anterior rhinoscopy revealed massive nasal polyps obstructing the cavum and vestibule of the nose on both sides (Figure 2). On a CT scan, a complete obliteration of the nose and paranasal sinuses as well as thinning and massive expansion of the nasal bone was seen (Figure 3). A comparison to the CT scan from 2002 was not possible. Since the picture data was archived for 10 years and then destroyed in 2012, the written radiologist's report describes no nasal bone thinning or expansion. The patient's test results with Sniffin' sticks again showed an anosmia (identification of 2 out of 12 possible odors). Medical treatment consisting of topical and systemic steroids failed and an endonasal endoscopic polypectomy and revision ethmoidectomy were performed in May 2014. Simultaneously, the bony nasal vault was narrowed by forced digital compression without osteotomies (Figure 4). The bone appeared to be thinned sufficiently to give in upon digital compression. Impression of the bony nasal sidewall resulted in a visible depression in the overlying skin which was corrected by a transcartilaginous decollement of the skin and subcutaneous tissues in a supraperiosteal plane. No nasal packing was inserted. A nasal cast was applied and left in place for one week.

**Figure 5:**
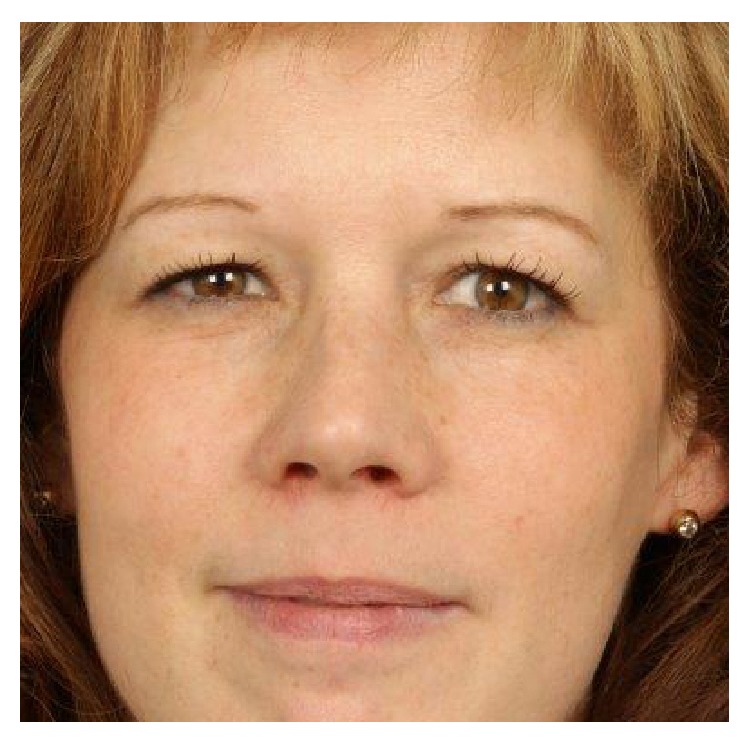
3 months after surgery.

Histology of the polyps again showed squamous epithelium and respiratory mucosa with light to medium signs of inflammation, some fibrosis, and severe eosinophilia. The patient reported a dramatically improved nasal air passage. A strict postoperative regimen including nasal saline irrigation and topical application of mometasone spray and fluticasone nasal drops was installed. At 3-month follow-up, minimal polypoid tissue was noted in the ethmoidal region, the nasal air passage remained free, and the patient reported a return of the sense of smell and was able to identify correctly all 12 possible odors in the Sniffin' sticks test. The form of the exterior nose remained stable (Figure 5). Again an aspirin desensitization was discussed but declined by the patient. The most recent follow-up in January 2015 (8 months after surgery) showed stable findings.

## 3. Discussion

Nasal polyps have been described since the times of Hippocrates and Galen [2]. Woakes first described a form of necrosing ethmoiditis and mucous polyps to the medical society of London in 1885. He also reported a “broadening of the bridge of the nose” in these cases that he found to be occurring “sometimes” [3]. In 1924, the term Woakes' syndrome was coined by Appaix and Robert [4] to describe a condition of bilateral nasal polyps beginning during childhood, ethmoiditis, and hypertrophic process with nasal pyramid deformation and therapeutic failure. Kellerhals and De Uthemann defined Woakes' syndrome in 1979 as the broadening of the nose, frontal sinus aplasia, bronchiectasis, and dyscrinia (production of highly viscous mucus) [5]. Appaix and Robert included patients with eosinophilic and noneosinophilic polyposis in their definition of Woakes' syndrome [4]. In recent years, Woakes' syndrome has usually been characterized as severe recurrent nasal polyps with consecutive destruction of the nasal pyramid leading to the broadening of the nose due to the chronic pressure of the polyps [1]. Most cases of Woakes' syndrome seem to occur in children and young adults [4–9] due to the plasticity of the developing and growing bony facial structures. The etiology remains unknown. Several authors have described cases of synchronous occurrence in siblings [5, 7], making heredity a potential contributing factor. The high incidence of nasal polyps in patient with aspirin intolerance has been well documented since Widal's first description in 1922 and Samter's coining of the term “asthma triad” in 1968 defined by combination of nasal and bronchial disease and aspirin sensitivity [10].

Our patient exhibited several risk factors for nasal polyps with a positive family history, aspirin intolerance, and multiple allergies. However, it remains unclear why some patients with nasal polyps develop a deformation of the nasal pyramid while most show no bone atrophy or only a deformity limited to the paranasal sinuses. This case documents the development of the condition over a period of more than 10 years during adulthood. In the years after the first surgical treatment, the recurrence of the nasal polyps led to a distinct deformation of the nose. Identical histological findings after both procedures fail to explain the change in biological behavior of the polyps leading to nasal deformity.

Our patient fulfills the criteria for Woakes' syndrome as defined by Appaix and Robert [4] even though a Samter's triad is probably the origin of the disease in this case.

Foze et al. described rhinoplasty as part of the treatment in Woakes' syndrome [9], but, apart from this description, treatment appears to be typically limited to endoscopic sinus surgery. In our case, the atrophy of the nasal bone appeared to be such that it could be fractured without osteotomy. This simple and efficient procedure resulted in an adjustment of the nasal form which was felt to be very satisfactory by the patient. This case illustrates the feasibility of a combined approach to correct the external nasal deformity during or after endoscopic sinus surgery.

## 4. Conclusion

Woakes' syndrome is well described in the literature although its exact etiology remains unclear. There is general consensus concerning the treatment of the nasal polyps by endoscopic sinus surgery. Topical and, if necessary, systemic treatment of the nose should be undertaken to slow down or avoid recurrence of the nasal polyps.

The treatment of the external nose should be addressed during the primary surgery if possible.

## References

[1] Caversaccio M., Baumann A., Helbling A. (2007). Woakes' syndrome and albinism. *Auris Nasus Larynx*.

[2] Hargrove S. W. (1954). Discussion on the treatment of nasal polypi. *Proceedings of the Royal Society of Medicine*.

[3] Woakes E. (1885). Necrosing ethmoditis and mucous polyps. *The Lancet*.

[4] Appaix S., Robert J. (1953). Polypose déformante et récidivante des jeunes (maladie de Woakes). *Revue de Laryngologie*.

[5] Kellerhals B., De Uthemann B. (1979). Woakes' syndrome: the problems of infantile nasal polyps. *International Journal of Pediatric Otorhinolaryngology*.

[6] Bailey D. L. (1965). Deformity of the nose and face due to nasal polyposis. *The West Indian Medical Journal*.

[7] Groman J. D., Bolger W., Brass-Ernst L., MacEk M., Zeitlin P., Cutting G. (2004). Recurrent and destructive nasal polyposis in 2 siblings: a possible case of Woakes' Syndrome. *Otolaryngology—Head and Neck Surgery*.

[8] Triglia J. M., Dessi P., Cannoni M., Pech A. (1992). Intranasal ethmoidectomy in nasal polyposis in children. Indications and results. *International Journal of Pediatric Otorhinolaryngology*.

[9] Foze A. N., Reynoso V. M., Deutsch Reiss E. (1987). Woake's syndrome. A case report in a teenager. *International Journal of Pediatric Otorhinolaryngology*.

[10] Picado C. (2002). Aspirin intolerance and nasal polyposis. *Current Allergy and Asthma Reports*.

